# Crosstalk between p38 MAPK and GR Signaling

**DOI:** 10.3390/ijms23063322

**Published:** 2022-03-19

**Authors:** Lisa Zeyen, Ole Morten Seternes, Ingvild Mikkola

**Affiliations:** Department of Pharmacy, UiT-The Arctic University of Norway, 9037 Tromsø, Norway; lisa.z.oyas@uit.no (L.Z.); ole-morten.seternes@uit.no (O.M.S.)

**Keywords:** p38, MAPK, signaling, inflammation, cancer, glucocorticoid receptor, crosslink, inhibitor, drug discovery, glucocorticoids

## Abstract

The p38 MAPK is a signaling pathway important for cells to respond to environmental and intracellular stress. Upon activation, the p38 kinase phosphorylates downstream effectors, which control the inflammatory response and coordinate fundamental cellular processes such as proliferation, apoptosis, and differentiation. Dysregulation of this signaling pathway has been linked to inflammatory diseases and cancer. Secretion of glucocorticoids (GCs) is a classical endocrine response to stress. The glucocorticoid receptor (GR) is the primary effector of GCs and plays an important role in the regulation of cell metabolism and immune response by influencing gene expression in response to hormone-dependent activation. Its ligands, the GCs or steroids, in natural or synthetic variation, are used as standard therapy for anti-inflammatory treatment, severe asthma, autoimmune diseases, and several types of cancer. Several years ago, the GR was identified as one of the downstream targets of p38, and, at the same time, it was shown that glucocorticoids could influence p38 signaling. In this review, we discuss the role of the crosstalk between the p38 and GR in the regulation of gene expression in response to steroids and comprehend the importance and potential of this interplay in future clinical applications.

## 1. Introduction

Some of the most distinct molecular features and essential parts of mammalian cells are the mitogen-activated protein kinase (MAPK) signaling pathways structured in a three-tire of kinases—a MAPK, a MAPK kinase (MAP2K), and a MAPK kinase kinase (MAP3K). These pathways allow the cell to react to different internal and external stimuli such as stress factors, hormones, and cytokines to regulate proliferation, differentiation, and apoptosis, among other fundamental processes. Dysfunctions due to mutations within the MAPK pathways have been correlated to several clinical outcomes and health issues like Alzheimer’s disease, Parkinson’s disease, inflammation, and multiple types of cancer [1,2,3]. Thus, a deepened insight into the mechanisms of MAPK signaling is of great interest when it comes to understanding the pathology and to developing new treatments for major human diseases.

The p38 MAPK pathway is one of three major MAPK signaling pathways present in human cells, next to the extracellular signal-regulated kinase (ERK) and c-Jun NH(2)-terminal kinase (JNK) MAPK pathways. There are five different kinases known to activate p38 signaling, the MAP3Ks ASK1/2 (MAP3K5/6), TAK1 (MAP3K7), TAO1 (MAPK16), and TAO2 (MAP3K17). Downstream of these are the two MAP2Ks, MKK3, and MKK6, and their target protein isoforms p38 MAPK α, β, γ, and δ [4,5]. The p38 kinase, and particularly the isoforms α and β, further activate several substrates like Ser/Thr activated protein kinases MK2/3, MNK1/2, and MSK1/2, as well as transcription factors like ATF2, leading to effective, context-dependent functions such as inflammatory response and cell apoptosis, as well as tumor-promoting or -suppressing effects [6,7]. 

Acknowledging its clinical value, the research on p38 has been intensified over the years and specific inhibitors mainly targeting p38α and -β are generated and characterized thoroughly. Early inhibitors like SB203580 and SB202190 act on the ATP binding site, while newer ones like BIRB 796/Doramapimod interfere indirectly with ATP binding, preventing p38’s catalytic activity. Nowadays, these tools are widely used for in vitro and in vivo studies, as well as being investigated in pre-clinical trials and offering new host-modulating therapy forms for various diseases including rheumatoid arthritis, Crohn’s disease, tuberculosis, and cancer [8,9].

The glucocorticoid receptor (GR) is expressed in all cells of the human body and its ligands, the glucocorticoids, display important functions within development, differentiation, and homeostasis. GR affects glucose and fat metabolism, the immune system, the nervous system, and the musculoskeletal system [10]. As a member of the nuclear receptor superfamily, GR influences and controls gene expression via hormone-dependent activation, causing conformational changes and translocation. The receptor cycles between the cytoplasm and nucleus are dependent on the ligand-bound and activation state. The modular receptor protein consists of an N-terminal transactivation domain (AF1), a central DNA-binding domain consisting of two zinc fingers, and a C-terminal ligand-binding and activation domain (AF2). GR can bind to specific sites in the genome either as a dimer or a monomer or interact with other transcription factors (TFs) to bind composite elements. It can also interact indirectly with DNA by binding TFs that are already bound to promoters (tethering) [11]. The DNA sequences responsive to GR are so-called glucocorticoid response elements (GREs) that are distributed widely throughout the genome. Gene regulation by GR is highly context-dependent and subject to the accessibility of chromatin, post-translational modifications, and association with other TFs and co-factors [11,12,13]. Due to alternative splicing and the use of alternative translational start codons, there are several receptor isoforms generated from the gene (*NR3C1*) encoding GR [14]. GRα and GRβ are the main isoforms, and they differ in their C-termini including the ligand-binding domain. GRα fulfills the classic GR transcriptional function upon activation via corticosteroids and is the isoform referred to when one talks about GR in general. The GRβ isoform lacks ligand binding activity and has been shown to reside in the nucleus where it counteracts the GRα function [15,16], but is also reported to have intrinsic activities regulating gene expression independent of GRα [17]. The way GR regulates transcription is complex, involving the cooperation of numerous transcription factors and co-factors, leading to both activation and repression of genes in a tissue-specific manner. Several post-translational modifications are also known to affect GR function [11].

The GR ligands, corticosteroids, and glucocorticoids (GCs) are steroid hormones released by the adrenal cortex that regulate major biological processes such as development, metabolism, and inflammation. Due to their immunosuppressive effects, synthetic GCs like dexamethasone are widely used in clinics for the treatment of autoimmune and inflammatory diseases [18,19]. They are the first-line treatment for lymphoid cancers causing apoptosis [20], whereas, for patients with solid tumors undergoing chemotherapy or radiation, they are used to treat side effects such as nausea, bone pain, and edema. However, an emerging problem regarding the GC treatment of cancer and other diseases like chronic obstructive pulmonary disease (COPD) and severe asthma, is the development of insensitivity and/or resistance, which prevents effective therapy [21]. 

In previous years of research, the molecular mechanisms regarding poor responsiveness towards GCs and a potential restoration have been rising topics of research, identifying potential roles of GRβ expression, viral infections, and MAPK signaling [22,23,24,25].

## 2. Crosstalk between p38 and GR

That the MAPK signaling pathways and glucocorticoid signaling could affect each other mutually was already reported more than 20 years ago. The use of dexamethasone showed to cause an inhibition of the MAPK pathway in mast cells [26], while MAPKs were concurrently identified as some of the kinases capable of phosphorylating GR at specific sites in vitro [27]. The first report of GCs inhibiting MAPK activity only involved ERK1/2 [26], however, they were soon reported to inhibit the stress-activated JNK and p38 signaling pathways as well [28,29]. Further on, the specificity of MAPK’s phosphorylation of GR was investigated, and JNK was shown to directly phosphorylate rat GR at Serine (S) 246, corresponding to human GR S226 [30]. Activated ERK2 was further characterized to indirectly affect this phosphorylation site, while p38 had no effect [30]. Since then, it has successfully been established that p38 can regulate phosphorylation of GR at specific sites as well. The activation of the p38 kinase and GR phosphorylation are shown to correlate within several diseases, and this interaction is thought to contribute to the GC resistance observed in the clinic. Below, we attempt to summarize the current research data concerning the crosstalk between the p38 and the GR signaling pathways and provide examples of corresponding biological consequences and the clinical relevance of this crosstalk.

### 2.1. p38-Directed GR Phosphorylation and Regulation

One of the first hints towards a clinically relevant crosstalk between p38 and GR signaling was the study by Irusen et al. [31], which demonstrated that the p38 inhibitor SB203580 potentially reverses the GC insensitivity in patients with severe asthma. The authors isolated peripheral blood mononuclear cells from asthma patients and could show that stimulation with a combination of IL2 and IL4 activated p38, which then resulted in increased GR phosphorylation. This phosphorylation and an altered ligand binding affinity could be inhibited when applying the p38 inhibitor SB203580, but not by an ERK1/2 pathway inhibitor [31]. 

In the following years of research, the development of phospho-specific antibodies made it easier to study individual GR phosphorylation sites, and the S211 phosphorylation (hGR) was suggested as a biomarker for GR activation in response to hormone treatment [32]. Additionally, S211 was identified as a direct substrate of p38 in lymphoid cells [33]. The application of p38 inhibitors hereby caused a decrease in dexamethasone-induced apoptosis, demonstrating a correlation between p38 activation and glucocorticoid-driven apoptosis [33]. The phosphorylation of S211 was also shown to play a part in GR’s regulation of carbohydrate metabolism. Here, the AMP-activated protein kinase (AMPK), induced by AICAR, was shown to activate p38, which then mediated the phosphorylation of GR at S211 [34]. This event caused tissue- and gene-specific alterations of GR-induced target gene expression by changing the attraction of transcriptional coregulators to DNA-bound GR [34]. The p38-mediated GR phosphorylation seems to vary greatly dependent on cell type, stimulus, and function. In airway smooth muscle (ASM) cells, inhibition of p38 activity decreased basal and fluticasone propionate-induced GR S203 phosphorylation, while increasing basal and fluticasone propionate-induced S211 phosphorylation [35]. Notably, no effect was observed on S226 phosphorylation. The p38-induced S203 phosphorylation had an inhibitory effect on GR translocation to the nucleus, GRE-reporter gene expression, and mRNA expression of the Glucocorticoid-induced leucine zipper (GILZ) gene. This was verified by the use of siRNA against p38, whereas co-transfection with a vector expressing a constitutive active MKK3 mutant activating p38 had the opposite effect. The usage of an S203A mutant hereby confirmed that the phosphorylation of this residue was inhibitory for GR function. The authors concluded that basal p38 MAPK activity in ASM cells was important to keep unliganded GR in an inactive state [35]. 

The residue S226 of GR was shown to be phosphorylated in vitro by both JNK and ERK2, but not by the p38 MAP kinase [30]. In these studies, the activation of either ERK1/2 or JNK resulted in the repression of GC-mediated transcription. However, a functional S226 was only required for JNK’s repression of GC-induced transcription. This phosphorylation seems to play an important part in the nuclear export of GR [36], and a few studies have also reported that p38 is responsible for the GR S226 phosphorylation in peripheral blood mononuclear cells derived from asthmatic patients. However, these observations are solely based on the quantitation of phospho-S226 western blots, where a minute reduction of S226 phosphorylation is observed in activated cells treated with p38 inhibitors [37,38,39]. Despite the convincing results regarding a connection between p38 activation and a general phosphorylation of GR in the cells and cell lines used, a direct connection between p38 and the GR S226 phosphorylation is not definitively proven. Additional experiments would be required before the S226 of GR can be confirmed as a bona fide p38 phosphorylation site. 

The phosphorylation of S134 in GR was shown to be dependent on p38 activity and independent of GC ligand binding [40]. It is shown to be induced by stressors such as H_2_O_2_, UV light, and hypoxia, and abolished by the use of a p38 inhibitor in various mammalian cells [40,41]. Furthermore, TGFβ1 can induce phosphorylation of S134 via activation of p38 in triple-negative breast cancer (TNBC) cells, and a functional S134 phospho-site was shown to be required for migration, growth in soft agar, and formation of tumor spheres [42]. Notably, the TGFβ1, as well as the stress-induced phosphorylation of S134, were independent of hormone stimulation, and the TGFβ1-stimulated S134 phosphorylation resulted in a positive feedback loop stimulating the p38 pathway [42]. Phosphorylation of S134 is further shown to enhance GR’s interaction with 14-3-3ζ and PELP1 and induce altered gene expression [40,41,42]. Interestingly, phosphorylation of S134 by AKT1 is reported to be inhibitory by preventing the nuclear translocation of GR [43], and the mechanism here is reported to be interaction with another member of the 14-3-3 family in the cytoplasm [44,45]. The application of an S134A mutant cell line additionally identified a specific TGFβ1-induced pS134 gene signature, indicating that this phosphorylation is necessary for the specific transcription of genes critical for MAPK signaling (e.g., MAP2K5) and cell migration [42]. 

**Figure 1:**
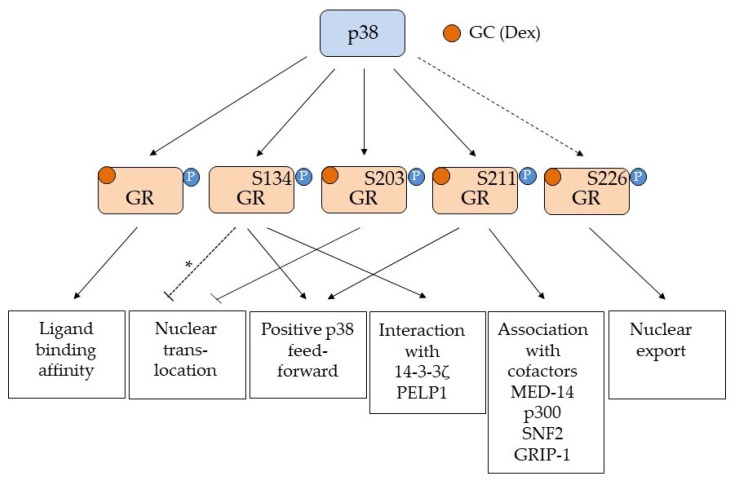
Overview of the GR phosphorylation sites affected by p38 MAPK. Serine (S) 134, 203 and 211 are all phosphorylation sites shown to be affected by p38 MAPK activation. Serine (S) 226 is a JNK phosphorylation site known to enhance nuclear export [36] and is indicated (but not convincingly proven) to be a p38 MAPK phospho site [37,38,39] (stippled line). The various responses to the individual p38 phosphorylation events are shown in the boxes below. Arrows indicate activation/positive regulation, while a blunted line indicates inhibition. The GR ligand is indicated as GC (glucocorticoid) and Dex (Dexamethasone). For S134, a phosphorylation by AKT1 is reported to prevent nuclear translocation [43], but since this is not proven to be the effect of p38 phosphorylation of this site, it is marked with a stippled line with an asterisk (*). p38-dependent phosphorylation of unspecified residues in GR affects ligand binding [31], while pS134 is shown to be ligand independent and to generate a positive feed forward loop with p38 [33] as well as promoting interaction with 14-3-3ζ [40,42] and PELP1 [41]. p38 dependent phosphorylation of S203 prevents nuclear translocation [35], while pS211 is associated with ligand bound and activated GR and are important for GR’s interaction with transcriptional co-factors such as MED-14 [49], p300 [34], SNF2 [34] and GRIP-1 [51]. GR pS211 will also contribute to a positive feed forward loop with p38 [42]. The sum of all these phosphorylation events will affect the transcriptional regulation exerted by GR. Importantly the phosphorylation of S203, S211 and S226 seem to influence each other, and the relationship between the phosphorylation events for pS203 and pS211 [32,35,51] and pS211 and pS226 [48,49] will contribute to the selection of genes transcribed by GR in a cell and tissue specific manner.

The p38-dependent phosphorylation of GR seems to be induced by different stressors like nutrient deprivation, H_2_O_2_, hypoxia, and ROS [34,40,41,42], as well as signals associated with inflammation such as LPS, IL2/IL4, and Ilα [31,46,47]. The outcome of this phosphorylation is cell- and tissue-specific and dependent on the phospho-site(s) affected. A summary of the effects of the individual p38-mediated GR phosphorylation events is given in Figure 1. The phosphorylated S211, in general, seems to be associated with the activation of GR, supported by the fact that this site is regulated in a ligand-dependent manner [32,33]. However, it is also shown that the proportion of phosphorylated S211 compared to both pS203 [32,35] and pS226 [48,49] influences GR activity, and that differential phosphorylation influences the structure and activity of the AF1 domain [50] and the interaction with the coactivator GRIP-1 [51]. S203 phosphorylation may prevent nuclear translocation and is associated with decreased GR activity [35], whereas S211 phosphorylation increases nuclear translocation and interaction with transcriptional cofactors [34,49], thus promoting GR-mediated transcription. The phosphorylation status of GR influences which promoters it is recruited to [48], and the p38-dependent phosphorylation of GR is also reported to affect ligand binding affinity [31] and binding to scaffolding proteins such as the 14-3-3 family [40,42]. 

Finally, it should be mentioned that the p38 MAP kinase may also affect GR activity by indirect mechanisms. GAL4DBD fusion experiments performed in HeLa cells showed that the negative effect that activated p38 had on GR activity depended on the LBD/AF2 region of GR, where there are no phosphorylation sites for p38 [52].

### 2.2. GR Regulated p38 MAPK Phosphorylation and Dephosphorylation

It is well known that GCs can influence the MAPK signaling pathways, both by genomic and non-genomic means, and that this contributes to their anti-inflammatory attributes [53,54]. Studies show that ERK1/2, JNK, and p38 MAPK phosphorylation and activation can be affected after stimulation with glucocorticoids. Although the mechanism of this regulation is not fully elucidated, the GC-mediated increase in transcription of the MAPK phosphatase 1/Dual-specificity phosphatase 1 *(MKP-1/DUSP-1*) seems to play a major part [55]. DUSP1 belongs to a family of phosphatases dedicated to an inhibitory regulation of MAP kinase activity by dephosphorylation of the threonine and tyrosine residues in their activation loop [56]. Glucocorticoids can induce the abundance of DUSP1 through induction of mRNA expression, as well as prevention of proteasomal protein degradation [55]. The MAP kinases JNK and p38 are major substrates of DUSP1, and GCs may negatively regulate the inflammatory effect of both the p38 and the JNK signaling pathway in macrophages via induction of DUSP1 expression [57,58]. 

However, there are also reports indicating that GCs not only repress MAPK signaling, but that they are able to stimulate p38 activity as well. One of these mechanisms involves a GR-dependent increase in expression of the upstream p38 activators *MKK3* [33] and *MAP3K5* [42]. Dexamethasone stimulation of several lymphoid cell lines hereby caused a modest increase in *MKK3* mRNA, and the positive effect on p38 activation was detected after 8 h or more. Notably, neither *MKK6* nor *MKK4* mRNA was affected [33]. Interestingly, in TNBC cell lines, upon stimulation with TGFβ1, unliganded GR was activated by a p38-mediated phosphorylation of S134 [42]. This activation caused an increase in both MAP3K5 mRNA and protein levels, and it was shown that the utilization of an S134A mutant cell line causes a decrease in MAP3K5 protein expression. The observations of these studies pointed towards the existence of a positive feed-forward loop between the p38 and the GR signaling pathways in TNBC cells [42].

Non-genomic and rapid effects of GCs on p38 MAPK activation have also been reported in several cell types [59,60,61]. In rat PC12 cells [59] and in rat primary cultured hippocampal cells [60], the GC-dependent activation of p38 was fast (within 5 min) and proved to be dependent on an active protein kinase C (PKC), since the PKC inhibitor Gø6976 would block this effect. Remarkably, the GR antagonist RU38486 (mifepristone) did not have any effect, indicating that GR is not involved in mediating this rapid GC-dependent activation of p38 in these cell types. However, in muscle cells, rapid activation of p38 by GCs has also been reported. In this case, the effect was mediated by GR localized in the membrane and extracellular matrix (ECM) [61] and was dependent on the muscle fiber type and the duration of the treatment. The GR antagonist RU38486 abolished this effect, as did a monoclonal antibody against the receptor, indicating that it was extracellularly located GR that mediated this. Since a Focal Adhesion Kinase inhibitor (FAK inhibitor 14) abolished the phosphorylation of p38, the signaling from GR to p38 seemed to go through FAK [61]. The authors also showed that GR can interact with laminin in the ECM, hypothesizing that GR being activated by GCs causes a structural change in laminin and an activation of FAK by integrins. 

Finally, GR is reported to activate p38 indirectly by causing an increase in reactive oxygen species (ROS). In chondrocytes, treatment with dexamethasone caused higher levels of NOX4 expression, which thereby increased ROS and led to activation of p38. This further affected the expression of matrix metalloproteinase-13 (MMP-13) and induced apoptosis [62].

### 2.3. Clinical Aspects of p38 and GR Crosstalk

After the confirmation of a direct crosstalk between the p38 MAP kinase and the glucocorticoid receptor, several studies have been focusing on the importance and practical usage of this interaction in the clinic. The interaction between p38 signaling and GR activity may be different depending on the diseases and tissues affected. Because GCs have immunosuppressive and anti-inflammatory functions, synthetic glucocorticoids (e.g., dexamethasone and prednisolone) are widely used for the treatment of chronic inflammatory conditions including respiratory, autoimmune, and cutaneous diseases [63]. Inhibition of the pro-inflammatory effects of p38 activity is one of the mechanisms used by GR to achieve this [54]. The p38 activation seems also to be involved in the induction of apoptosis caused by GCs in leukemic cancer cells [33] and in chondrocytes [62]. However, a major problem with the use of GC in the clinic is GC resistance or decreased GC sensitivity, and several examples indicate that p38 activity contributes to this resistance [38,39,64]. For an extensive review on MAPKs (also including ERK and JNK) and their involvement in GC resistance, the reader is referred to [22]. GCs are the preferred therapy for asthma patients, and corticosteroid insensitivity, leading to the development of severe asthma and poor life quality of the patients, is one of the major limiting factors for successful treatment and prognosis [25,31]. Relevant data of patients with severe asthma suggested that p38 activation leads to the phosphorylation and inactivation of GR, followed by subsequent corticosteroid insensitivity. The application of a p38 inhibitor can restore GC sensitivity and has been shown in peripheral blood mononuclear and bronchial epithelial cells derived from patients with severe asthma and chronic obstructive pulmonary disease (COPD) [38,39,64]. The combination of p38 inhibitor and dexamethasone is also shown to have a beneficial effect unrelated to the restoration of GC sensitivity. This was illustrated in a recent study, where an additive anti-inflammatory effect was observed in a TNF-α stimulated human lung fibroblast cell line and in fibroblasts obtained from human lung tissue [65]. For patients with cystic fibrosis (CF), where GCs are used as anti-inflammatory drugs to reduce lung inflammation, occurring insensitivity is also a problem. Here again, treatment with p38 inhibitors can increase the responsiveness to glucocorticoids in CF bronchial epithelial cells [66]. 

The crosstalk between p38 and GR was also shown for conditions like neuropathic pain and pancreatitis. In the spinal cords of rats with spared nerve injury, activation of p38 leads to the downregulation of GR expression, activation of NF-κB signaling and the generation of an inflammatory reaction and neuropathic pain [67]. Furthermore, dexamethasone has been shown to inhibit p38 activity in mild but not in severe acute pancreatitis [68], and hydrocortisone was recently shown to inhibit the observed upregulation and activity of p38 evoked by a co-culture of pancreatic acinar and stellate cells [69]. Achieving deeper insights into the crosstalk between GR and p38 might therefore not only provide means to combat GC resistance but may also provide new therapy options for diseases like neuropathy and acute pancreatitis in the future.

### 2.4. Cancer-Related Interactions

Glucocorticoids are used as a first-line treatment to induce apoptosis in lymphoid cancers [20] but are also widely applied to treat secondary effects caused by chemotherapy and irradiation in various solid tumors [70,71].

It has long been known that activation of the p38 pathway has been linked to the GC-induced apoptosis in lymphoid cells [33]. In contrast to the treatment of asthma, where p38 activity is inhibited for the restoration of GC function, GC resistance in acute lymphoblastic leukemia (ALL) is relieved by activation of the p38 pathway. Anisomycin, a potent p38/JNK signaling activator, has for instance when used in a low dose in combination with dexamethasone been reported to sensitize the GC-resistant T cell leukemia cell line CEM-C1 [72]. This co-treatment activated GR, JNK, and p38, all of which were inhibited using GR antagonist RU486, suggesting that GR might be upstream of the p38/JNK pathway in this cell line [72].

The effect of GC treatment on solid tumors is disputed. While there are studies showing that utilization of GCs and/or high expression of GR has no effect on tumor development [73] or even a positive effect [74,75], several reports indicate a negative outcome correlated with poor prognosis and increased risk of metastasis in the patients [76,77,78,79]. Several papers by Carol Lange’s research group have shown that p38’s phosphorylation of S134 is more prominent in triple-negative breast cancer (TNBC) than in other breast cancer subtypes and that this phosphorylation mediates the GR/HIF-1 interaction and induced expression of the breast tumor kinase *PTK6/Brk* gene, which is associated with aggressive breast cancer development [41,80] Further, this group has shown that TGFβ1 is able to activate p38 and phosphorylate S134 and that this phosphorylation is essential for survival, migration, invasion, and stemness properties of the TNBC cancer cells [42]. GCs can interfere with chemotherapeutic treatment [81], and several of those treatments are able to activate the p38 pathway [82,83]. There are indications that the crosstalk between GR and p38 may play a part in chemotherapy resistance [80], and this should be followed up in future investigations.

## 3. Conclusions

The glucocorticoid receptor and the p38 kinase are both key mediators of mammalian stress responses. GR is directly regulated by the binding of stress hormones, the glucocorticoids, while p38 is regulated as part of a three-tier kinase cascade dependent on input from other stress-sensing receptors and molecules. More and more data indicate that these pathways do not operate independent of each other and that they in fact interact with and influence each other’s actions. GR mediates the regulation of genes that are either positive or negative regulators of the p38 signaling pathway, while p38, on the other hand, can mediate the phosphorylation of GR, influencing its localization and activity. The effects of activation of the p38 signaling pathway on GR-mediated transcription seems to clearly be cell- and tissue-specific. For example, a lack of inhibition of p38 activity by GC in asthmatic cells will result in GC resistance, while a failure of GC to induce activation of p38 will result in treatment resistance in leukemic cells. Thus, in order to treat this GC resistance, inhibition of p38 activity is required for asthmatic cells, whereas in leukemic cells, activation of p38 activity is beneficial.

In retrospect of the herein reviewed data, it can be summarized that the fine-tuned regulation of the stress response through both glucocorticoids and activation of the p38 pathway is important and that dysregulation of this response may be a consequence and cause of a wide range of diseases. Since the p38-GR crosstalk and its molecular effects are cell- and tissue-specific, detailed knowledge of the cell type specific interplay between these two stress-induced signaling pathways will be required for pinpointing the most optimal treatment strategies in the future. 

## Data Availability

Not applicable.
